# Rapid Visual Detection of *Pythium insidiosum* by LAMP-LFD

**DOI:** 10.3390/jof12080589

**Published:** 2026-08-08

**Authors:** Thanawat Sridapan, Atisak Jiaranaikulwanich, Chompoonek Yurayart, Theerapong Krajaejun

**Affiliations:** 1 Division of Microbiology and Immunology, Department of Preclinical Science, Faculty of Medicine, Thammasat University, Pathum Thani 12120, Thailand; 2 Department of Pathology, Faculty of Medicine, Ramathibodi Hospital, Mahidol University, Bangkok 10400, Thailand; 3 Department of Microbiology and Immunology, Faculty of Veterinary Medicine, Kasetsart University, Bangkok 10900, Thailand

**Keywords:** *Pythium insidiosum*, pythiosis, diagnosis, LAMP, lateral flow dipstick

## Abstract

*Pythium insidiosum*, the causative agent of pythiosis, remains an important pathogen in tropical and subtropical regions, where delayed diagnosis is associated with severe disease and poor clinical outcomes. We developed a loop-mediated isothermal amplification coupled with lateral flow dipstick (LAMP-LFD) assay for the rapid detection of *P. insidiosum*. The assay employs dual-labeled LAMP amplicons that are directly detected by a lateral flow dipstick, enabling simple visual interpretation without agarose gel electrophoresis. Analytical performance was evaluated using genomic DNA from 102 clinically relevant isolates, comprising 51 *P. insidiosum* isolates and 51 non-target organisms. The LAMP-LFD assay correctly detected all *P. insidiosum* isolates, achieving an inclusivity of 100.0% (95% CI: 93.0–100.0%), and correctly excluded 48 of 51 non-target organisms, yielding an exclusivity of 94.1% (95% CI: 83.8–98.8%). The assay demonstrated a detection limit of 10 fg of genomic DNA per reaction, representing at least 1000-fold greater analytical sensitivity than the reference multiplex PCR (m-PCR) assay. Clinical validation using 47 blinded animal tissue specimens showed a sensitivity of 90.3%, specificity of 87.5%, accuracy of 89.4%, and good agreement with the reference standard (culture and ITS sequence analysis) (κ = 0.76). In comparison, the m-PCR assay achieved a sensitivity of only 41.9%, indicating substantially lower clinical detection performance. In conclusion, direct LFD-based detection preserves the analytical performance of the established *P. insidiosum* LAMP assay while providing a clear and easily interpretable visual endpoint. The LAMP-LFD assay represents a rapid, sensitive, and practical tool for screening pythiosis, offering improved diagnostic performance and more reliable result interpretation.

## 1. Introduction

*Pythium insidiosum* is a fungus-like oomycete pathogen commonly found in swampy environments throughout tropical and subtropical regions worldwide [1,2]. It is the causative agent of pythiosis, a severe and potentially fatal disease affecting humans and a wide range of animals. In Thailand, although pythiosis has long been recognized as an important human disease, an increasing number of cases have recently been reported in animals, particularly dogs and horses [3,4,5]. The disease is characterized by aggressive progression, limited responsiveness to conventional antifungal agents, and poor prognosis [1,6,7]. Although awareness of pythiosis and research attention have increased over the past decade, the disease likely remains underdiagnosed in many regions [2,8]. Delayed diagnosis frequently results in postponement of appropriate treatment, thereby increasing the likelihood of extensive surgical intervention, including limb amputation and eye enucleation [9,10,11]. Therefore, timely and accurate diagnosis of pythiosis is essential for improving patient outcomes. Currently, diagnostic approaches for pythiosis include conventional culture, immunological assays, and nucleic acid-based tests (NATs) [12]. NATs offer rapid and specific detection of *P. insidiosum* and can support the definitive diagnosis of pythiosis [13,14]. As a result, they have been increasingly adopted in clinical laboratories for the rapid and reliable detection of *P. insidiosum*.

Loop-mediated isothermal amplification (LAMP) is an increasingly popular NAT that amplifies target DNA under isothermal conditions (60–65 °C) using *Bacillus stearothermophilus* (*Bst*) DNA polymerase and multiple primers targeting distinct regions of the target sequence [15,16]. Owing to its simplicity, rapidity, and high amplification efficiency, LAMP has been widely applied to pathogen detection, including *P. insidiosum* [17,18,19,20,21,22,23]. LAMP amplicons can be detected by turbidity measurement, agarose gel electrophoresis, or colorimetric assays [17,18,24,25,26,27]. However, each method has inherent limitations. Turbidity-based detection requires a dedicated spectrophotometric reader [28], whereas agarose gel electrophoresis is labor-intensive and prolongs turnaround time [17,29]. In contrast, colorimetric assays enable rapid visual interpretation without specialized equipment [30]. However, the results are often subjective and prone to misinterpretation, particularly when color changes are weak or borderline. Furthermore, substantial interobserver variation in the interpretation of results has been reported between colorimetric assays and gel electrophoresis, potentially compromising diagnostic reliability [23,31,32]. These limitations also apply to currently available *P. insidiosum*-specific LAMP assays [18,19,20], highlighting the need for a more reliable and user-friendly detection platform.

To overcome these limitations, lateral flow dipstick (LFD) technology has emerged as an attractive reporting platform for LAMP because of its low cost, rapidity, and ease of use [17,33,34,35]. LFD enables direct visual detection of double-labeled LAMP amplicons through gold nanoparticle-based signal generation, producing an easily interpretable test line [36,37]. As a direct readout method for LAMP amplicons, LFD provides clear visual results that are readily interpreted and comparable to those obtained by agarose gel electrophoresis [38,39,40,41]. Several studies have successfully applied LFD for LAMP-based diagnostics [17,33,34]. To date, no study has reported the use of a LAMP-LFD platform for detecting *P. insidiosum*. Therefore, in the present study, we developed a LAMP-LFD assay for the rapid detection of *P. insidiosum* and evaluated its sensitivity and specificity against a well-established multiplex PCR (m-PCR) assay [42]. Performance was assessed using purified DNA from *P. insidiosum* and various fungal controls, as well as blinded clinical specimens.

## 2. Materials and Methods

### 2.1. Microorganisms and DNA Extraction

A total of 102 clinically relevant organisms, comprising 51 *P. insidiosum* isolates and 51 non-target organisms, including three closely related *Pythium* species and 48 fungal isolates, were included to evaluate the detection performance of the LAMP-LFD assay (Table 1). The non-target organisms included one ATCC reference strain (*Pythium aphanidermatum* ATCC 32230), two environmental isolates (*Pythium catenulatum* and *Pythium rhizo-oryzae*), and fungal isolates maintained in our Mycology Unit culture collection, many of which were originally recovered from clinical specimens. All isolates were identified prior to inclusion in this study based on culture characteristics, morphological examination, and/or internal transcribed spacer (ITS) sequence analysis.

Genomic DNA was extracted from actively growing cultures of all organisms using a high-salt extraction method, as previously described [43,44]. Briefly, harvested hyphae were mechanically disrupted using glass beads (Sigma-Aldrich, St. Louis, MO, USA) and a TissueLyser MM301 mixer mill (Qiagen, Hilden, Germany), followed by lysis with SDS and proteinase K at 56 °C overnight. The lysate was treated with 6 M NaCl and centrifuged to remove cellular debris and proteins, and the DNA-containing supernatant was collected. Genomic DNA was precipitated with an equal volume of isopropanol and pelleted by centrifugation. The DNA pellet was washed with 70% ethanol, air-dried, and resuspended in 100 μL TE buffer. All extracted DNA samples were stored at −20 °C until use.

### 2.2. LAMP Primer Set and Labeling Strategy

A set of six primers previously described by our group [22] was used in this study. The primer set comprised two outer primers (F3 and B3), two inner primers (FIP and BIP), and two loop primers (LF and LB), which recognize eight distinct regions within the internal transcribed spacer (ITS) region of the ribosomal DNA (rDNA) of *P. insidiosum*. For LFD detection, the BIP-Biotin and LB-FITC primers were labeled at the 5′ end with biotin and fluorescein isothiocyanate (FITC), respectively. The primer sequences were as follows: F3, 5′-GGCAGAATGTGAGGTGTCTC-3′; B3, 5′-GGAAACAACACCCCGTCAG-3′; FIP, 5′-ACAGATCACTGCGTTCGAGCATTTTTGGAGATAGCACGAGTCCCT-3′; BIP, Biotin-5′-TCAGATTGCTTTGCGCTGGTGGTTTTCCGAAGCCTAACATACCGC-3′; LF, 5′-GACACAAGAGAGATCAACGTACATT-3′; and LB, FITC-5′-AGGACATTAAGGAGATGACCTCTAT-3′. All oligonucleotide primers were synthesized by Bio Basic Inc. (Markham, ON, Canada).

### 2.3. LAMP Amplification and LFD Detection

LAMP amplification was performed as previously described [22]. The reaction mixture comprised 1× ThermoPol Reaction Buffer, 6 mM MgSO_4_, 8 U *Bst* DNA polymerase (large fragment), 1.4 mM dNTPs (New England Biolabs Inc., Ipswich, MA, USA), 0.4 M betaine (Sigma-Aldrich, St. Louis, MO, USA), 0.2 μM each of primers F3 and B3, 1.6 μM each of primers FIP and BIP, 0.4 μM each of primers LF and LB, and 2 μL of template DNA. Sterile distilled water was added to a final volume of 25 μL. Amplification was carried out at 65 °C for 60 min, followed by enzyme inactivation at 85 °C for 5 min using a Mastercycler Nexus Gradient thermocycler (Eppendorf, Hamburg, Germany). Nuclease-free water was used as the no-template control. Following amplification, 1 μL of the LAMP product was mixed with 120 μL of running buffer (PBS containing Surfynol 465 surfactant; Kestrel Bio Sciences, Pathum Thani, Thailand). The LFD strip was then immersed in the mixture for 15 min. A positive result was indicated by the appearance of reddish-purple bands at both the test (T) and control (C) lines, whereas a negative result was indicated by the presence of a band only at the C line [33,45,46]. To verify successful amplification, 5 μL of the LAMP products were analyzed by electrophoresis on a 1.5% agarose gel stained with SERVA DNA Stain G (SERVA Electrophoresis GmbH, Heidelberg, Germany) and visualized under ultraviolet light using a UV transilluminator (Bio-Rad, Hercules, CA, USA). A characteristic ladder-like banding pattern was considered indicative of a positive LAMP reaction.

### 2.4. Multiplex PCR Assay

The m-PCR assay previously described by Rujirawat et al. [42] was used as a comparator molecular assay for evaluating the performance of the LAMP-LFD assay. For clinical specimen analysis, the diagnostic performance of both molecular assays was evaluated against the established reference standard comprising culture and ITS sequence analysis. The reaction mixture comprised 1× *Taq* buffer with KCl, 2 mM MgCl_2_, 0.2 mM dNTPs, 0.75 U *Taq* DNA polymerase (Thermo Fisher Scientific, Waltham, MA, USA), 0.12 μM primer ITS1 (5′-TCCGTAGGTGAACCTGCGG-3′), 0.07 μM each of primers R1 (5′-CCTCACATTCTGCCATCTCG-3′), R2 (5′-ATACCGCCAATAGAGGTCAT-3′), and R3 (5′-TTACCCGAAGGCGTCAAAGA-3′), and 2 μL of template DNA. Sterile distilled water was added to a final volume of 25 μL. Nuclease-free water was used as no-template control. PCR amplification was performed using a Mastercycler Nexus Gradient thermocycler (Eppendorf, Hamburg, Germany) under the following conditions: an initial denaturation at 95 °C for 5 min; 30 cycles of denaturation at 95 °C for 30 s, annealing at 59 °C for 30 s, and extension at 72 °C for 45 s; followed by a final extension at 72 °C for 10 min. PCR products were analyzed by agarose gel electrophoresis as described above.

### 2.5. Analytical Specificity by Inclusivity and Exclusivity Testing

The analytical specificity of the LAMP-LFD assay was evaluated through inclusivity and exclusivity testing. Inclusivity was assessed using 51 *P. insidiosum* isolates, whereas exclusivity was evaluated using 51 non-target organisms under the reaction conditions described above. Analytical specificity was determined based on the ability of the assay to correctly identify *P. insidiosum* isolates as positive and non-*P. insidiosum* organisms as negative. The experiment was independently carried out twice.

### 2.6. Analytical Sensitivity Assessment Using Reference Strains

The analytical sensitivity of the LAMP-LFD and m-PCR assays was assessed by determining their limits of detection (LODs). Ten-fold serial dilutions of genomic DNA extracted from three *P. insidiosum* reference strains (CBS573.85, Pi-S, and MCC13, representing clades I, II, and III, respectively) were prepared in sterile distilled water at concentrations ranging from 5 ng/µL to 0.5 fg/µL. The LOD was defined as the lowest DNA concentration that consistently yielded a positive result. The experiment was independently carried out twice.

### 2.7. Evaluation of LAMP-LFD and m-PCR Assays Using Clinical Specimens

A total of 47 frozen animal tissue specimens were obtained from the Mycology Unit, Department of Microbiology and Immunology, Faculty of Veterinary Medicine, Kasetsart University, Bangkok, Thailand. The specimens comprised tissues collected from 23 dogs, 22 horses, one cat, and one bird. Of these, 31 were obtained from animals diagnosed with pythiosis, whereas 16 were collected from animals without pythiosis and served as controls. All specimens were previously confirmed by culture and ITS sequencing. Culture and ITS sequence analysis were considered the reference standard for determining specimen classification throughout the study. Genomic DNA was extracted from the tissue specimens by mechanical grinding in liquid nitrogen, as described by Ferrer et al. [44], with minor modifications. The extracted genomic DNA samples were analyzed using the LAMP-LFD assay and compared with the results obtained using the m-PCR assay. All specimens were blinded prior to testing to minimize diagnostic bias during result interpretation.

### 2.8. Statistical Analysis

The diagnostic performances of the LAMP-LFD and m-PCR assays were evaluated by calculating sensitivity, specificity, positive predictive value (PPV), negative predictive value (NPV), and accuracy, along with their corresponding 95% confidence intervals (95% CIs), using MedCalc (https://www.medcalc.org/calc/diagnostic_test.php; accessed on 18 May 2026). Agreement between the two assays was assessed using Cohen’s kappa coefficient (κ). Kappa values were interpreted as poor (κ < 0.20), fair (0.21 ≤ κ ≤ 0.40), moderate (0.41 ≤ κ ≤ 0.60), good (0.61 ≤ κ ≤ 0.80), or very good (0.81 ≤ κ ≤ 1.00) agreement (https://www.medcalc.org/calc/kappa.php; accessed on 18 May 2026).

## 3. Results

### 3.1. Principle and Optimization of LAMP-LFD for Detecting P. insidiosum

**Figure 1:**
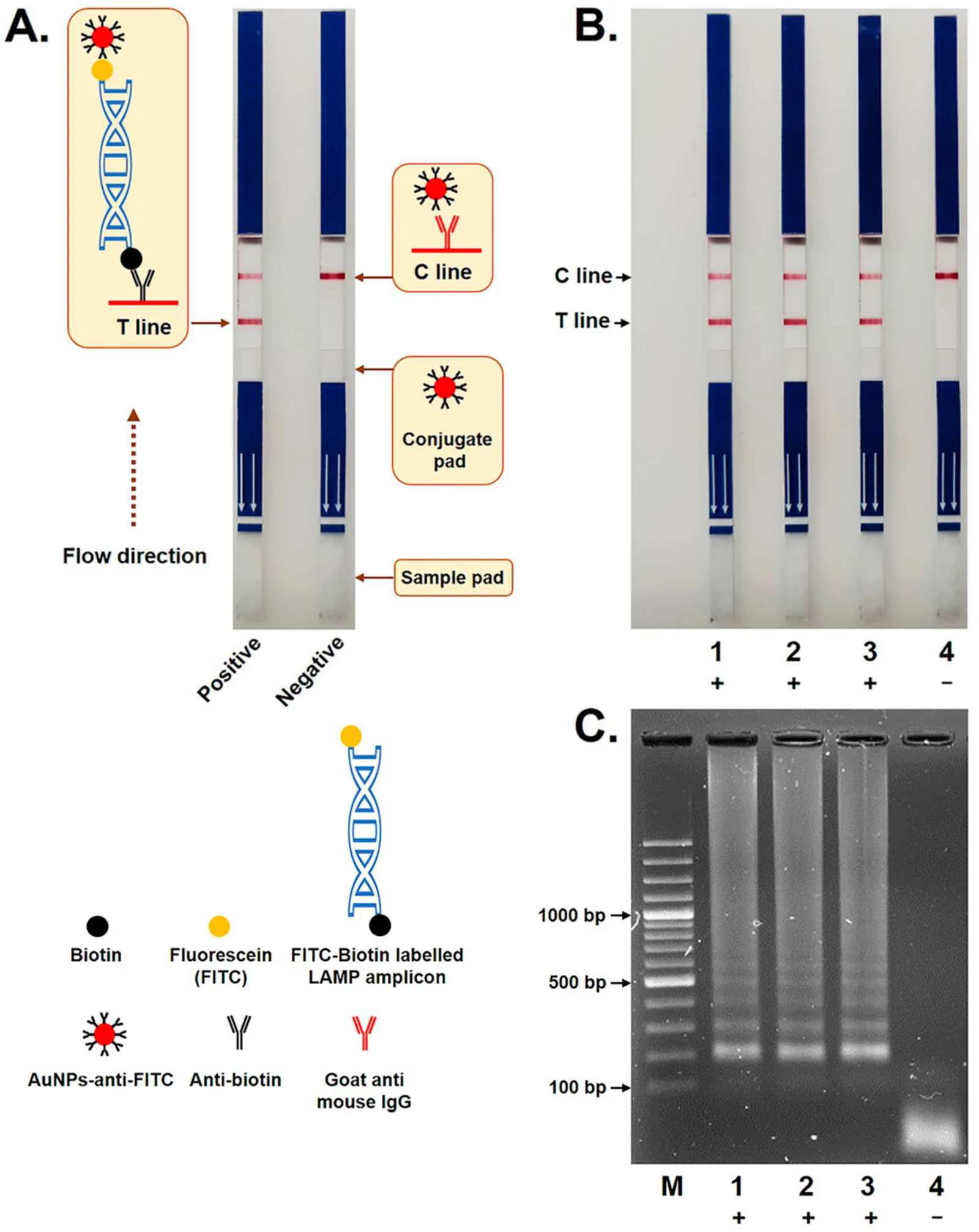
Principle and validation of the LAMP-LFD assay for the detection of *P. insidiosum*. (**A**) Schematic illustration of LAMP-LFD detection. FITC- and biotin-labeled LAMP amplicons bind to gold nanoparticle-conjugated anti-FITC antibodies (AuNPs-anti-FITC) and migrate along the lateral flow dipstick (LFD) by capillary action. The resulting complexes are captured by immobilized anti-biotin antibodies at the test (T) line, generating a visible red signal. Excess AuNPs-anti-FITC antibodies are captured by goat anti-mouse IgG antibodies at the control (C) line, confirming a valid test result. (**B**) Representative LFD results. Positive *P. insidiosum* samples are indicated by the presence of both T and C lines, whereas the negative control (no-template control) shows only the C line. (**C**) Agarose gel electrophoresis of the corresponding LAMP products. Positive reactions are characterized by the typical ladder-like banding pattern. In both the LAMP-LFD assay and agarose gel analysis, lanes 1–3 correspond to *P. insidiosum* strains CBS573.85 (clade I), Pi-S (clade II), and MCC13 (clade III), respectively, whereas lane 4 corresponds to the no-template control. Lane M indicates the 100 bp DNA ladder.

The LAMP-LFD assay was successfully optimized for the detection of *P. insidiosum* DNA using FITC- and biotin-labeled primers that generated double-tagged LAMP amplicons for subsequent lateral flow dipstick detection. As illustrated in Figure 1A, FITC-biotin-labeled amplicons bound to gold nanoparticle-conjugated anti-FITC antibodies and migrated along the LFD strip by capillary action. The resulting complexes were captured by immobilized anti-biotin antibodies at the T line, producing a visible red signal, while excess gold-conjugated antibodies were captured at the C line to confirm a valid test result. All three reference strains of *P. insidiosum* representing clades I, II, and III generated clearly visible bands at both the T and C lines of the LFD strip, whereas the no-template control produced a band only at the C line (Figure 1B). These findings were consistent with agarose gel electrophoresis, in which positive samples exhibited the characteristic ladder-like LAMP banding pattern and the no-template control showed no amplification products (Figure 1C).

### 3.2. Analytical Performance of LAMP-LFD

**Figure 2:**
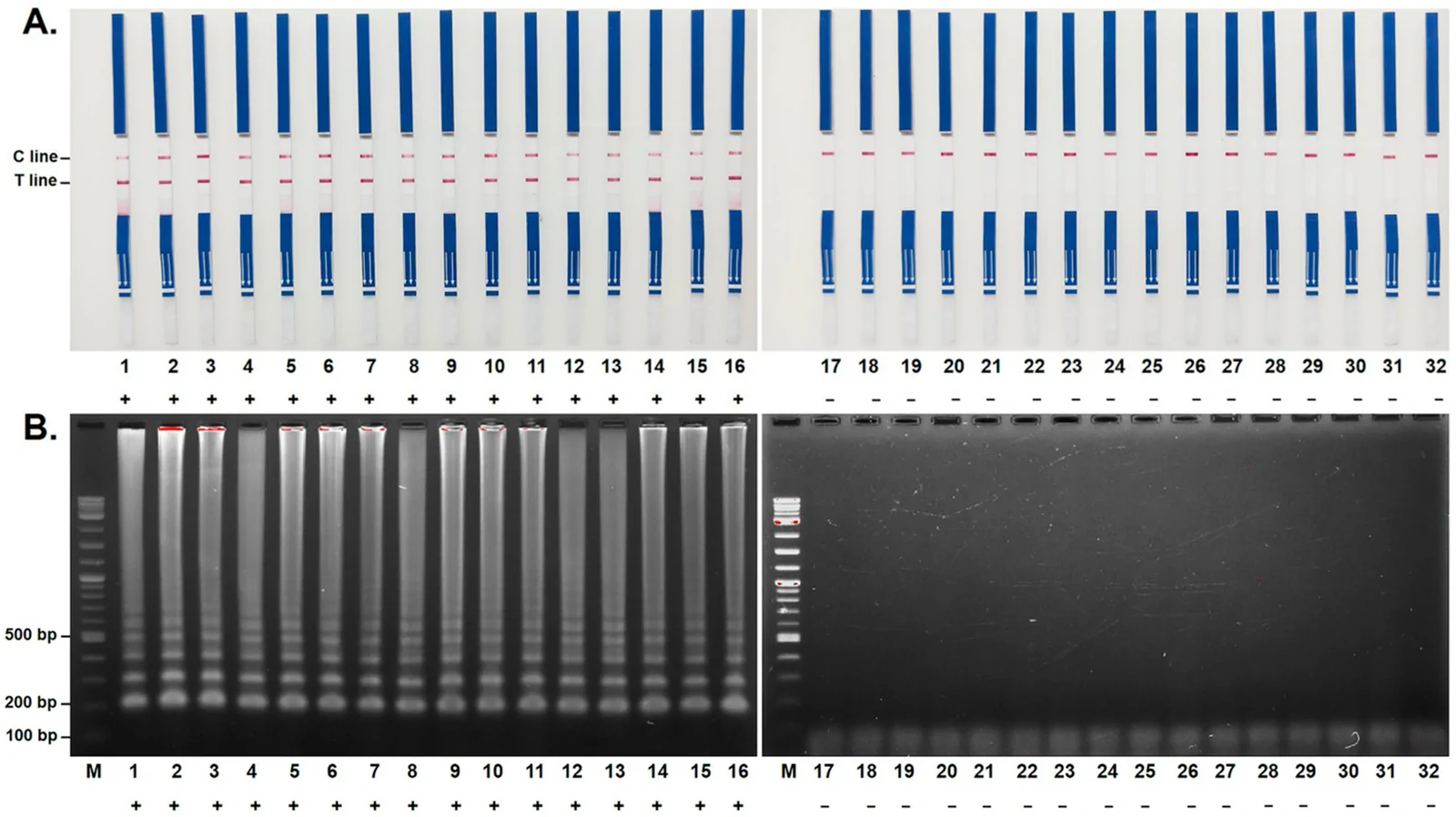
Analytical specificity of the LAMP-LFD assay. (**A**) Detection of representative *P. insidiosum* isolates, closely related *Pythium* species, and non-target fungal isolates using the LAMP-LFD assay. Positive reactions are indicated by the presence of both test (T) and control (C) lines on the LFD, whereas negative reactions are indicated by the presence of only the C line. (**B**) Agarose gel electrophoresis of the corresponding LAMP products. Positive reactions are indicated by the characteristic ladder-like banding pattern, whereas negative reactions are indicated by the absence of ladder-like bands. In both the LAMP-LFD assay and agarose gel analysis, lanes 1–13 correspond to *P. insidiosum* strains CBS573.85, P45-Br, EQ02, ATCC200269, Pi-S, P38, RT01, ATCC64221, MCC13, MCC17, KCB03, KCB09, and ATCC90586, respectively; lane 14 corresponds to *P. aphanidermatum* ATCC32230; lane 15 corresponds to *P. catenulatum* RM9-06; lane 16 corresponds to *P. rhizo-oryzae* RCB01; lanes 17–31 correspond to representative non-target fungal isolates; and lane 32 corresponds to the no-template control. Lane M indicates the 100 bp DNA ladder.

The analytical performance of the LAMP-LFD assay was assessed by evaluating its analytical specificity and analytical sensitivity. To evaluate the analytical specificity of the LAMP-LFD assay, genomic DNA from 51 *P. insidiosum* isolates, three closely related *Pythium* species, and 48 clinically relevant non-target fungal isolates was tested under the optimized reaction conditions. The LAMP-LFD assay successfully detected all 51 *P. insidiosum* isolates. Among the 51 non-target organisms tested, 48 were correctly identified as negative, whereas positive reactions were observed for three closely related *Pythium* species, namely *P. aphanidermatum*, *P. catenulatum*, and *P. rhizo-oryzae*, indicating cross-reactivity with these organisms (Table 1; Figure 2). Overall, the LAMP-LFD assay achieved an inclusivity of 100% (95% CI: 93.0–100.0%) and an exclusivity of 94.1% (95% CI: 83.8–98.8%). In contrast, the m-PCR assay correctly identified all 51 *P. insidiosum* isolates and showed no cross-reactivity with either the three closely related *Pythium* species or the 48 non-target fungal isolates (Table 1; Supplementary Figure S1).

**Figure 3:**
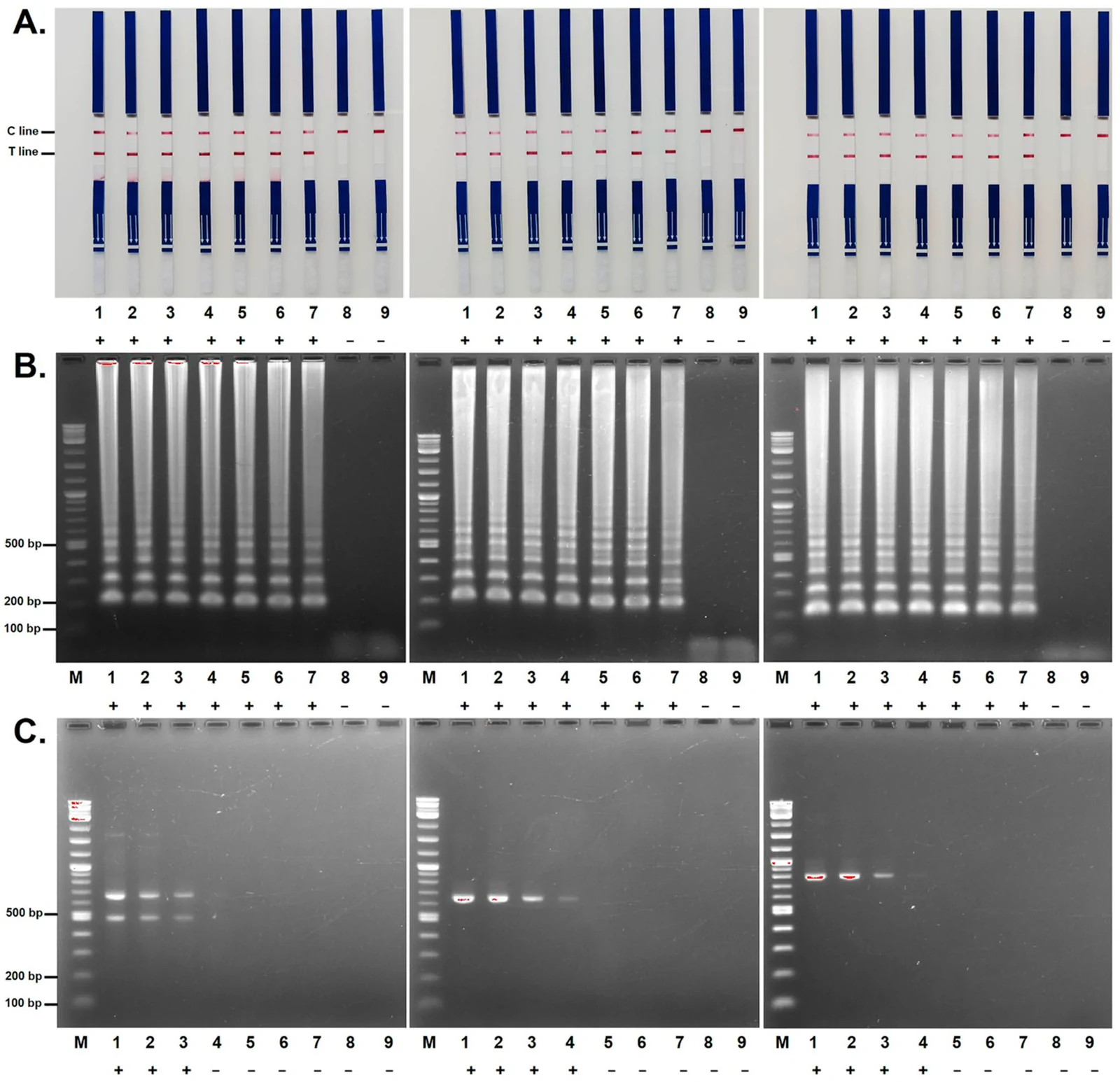
Analytical sensitivity of the LAMP-LFD and m-PCR assays for detecting *P. insidiosum* genomic DNA. (**A**) Lateral flow dipstick (LFD) detection of LAMP amplicons generated from 10-fold serial dilutions of *P. insidiosum* genomic DNA. (**B**) Agarose gel electrophoresis of the corresponding LAMP products. (**C**) Agarose gel electrophoresis of m-PCR products. In panels A–C, lanes 1–8 correspond to 10-fold serial dilutions of *P. insidiosum* genomic DNA at 10 ng, 1 ng, 100 pg, 10 pg, 1 pg, 100 fg, 10 fg, and 1 fg per reaction, respectively; lane 9 corresponds to the no-template control. Positive LAMP reactions are indicated by the presence of both test (T) and control (C) lines on the LFD or by the characteristic ladder-like banding pattern on agarose gel electrophoresis. Lane M indicates the 100 bp DNA ladder.

To determine analytical sensitivity, 10-fold serial dilutions of *P. insidiosum* genomic DNA ranging from 10 ng to 1 fg per reaction were evaluated using the LAMP-LFD assay. The LAMP-LFD assay consistently detected as little as 10 fg of genomic DNA from all three *P. insidiosum* reference strains representing clades I, II, and III, as determined by both LFD analysis (Figure 3A) and agarose gel electrophoresis (Figure 3B). No amplification was detected at lower DNA concentrations. In contrast, the m-PCR assay detected genomic DNA down to 10 pg per reaction for clade II and 100 pg per reaction for clades I and III (Figure 3C). The m-PCR assay also generated distinct amplification profiles that differentiated the three *P. insidiosum* clades, with 490 bp and 660 bp amplicons observed for clade I, a 660 bp amplicon for clade II, and an 800 bp amplicon for clade III. Based on the respective detection limits, the LAMP-LFD assay exhibited 1000- to 10,000-fold greater analytical sensitivity than the m-PCR assay for the detection of *P. insidiosum* DNA.

### 3.3. Evaluation of LAMP-LFD Using Clinical Specimens

**Figure 4:**
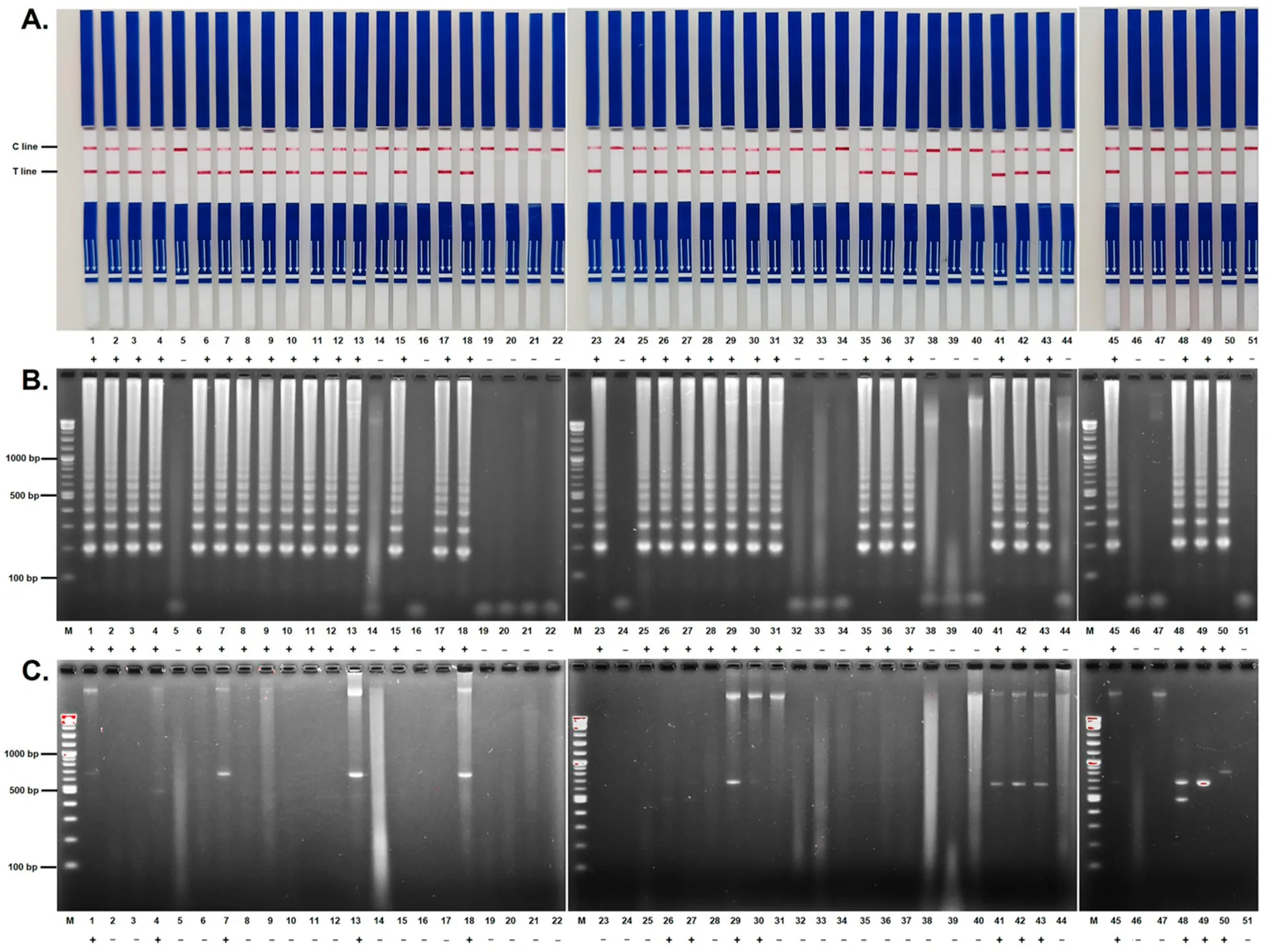
Detection of *P. insidiosum* in blinded clinical specimens using the LAMP-LFD and m-PCR assays. (**A**) LFD-based detection of LAMP amplicons obtained from 47 blinded clinical specimens. Positive reactions are indicated by the presence of both test (T) and control (C) lines, whereas negative reactions are indicated by the presence of only the C line. (**B**) Agarose gel electrophoresis of the corresponding LAMP products. Positive reactions are characterized by the typical ladder-like banding pattern. (**C**) Agarose gel electrophoresis of m-PCR products obtained from the same specimens. Lanes 1–47 correspond to individual clinical specimens. Lanes 48–50 correspond to positive controls comprising genomic DNA from *P. insidiosum* strains CBS573.85 (clade I), Pi-S (clade II), and MCC13 (clade III), respectively. Lane 51 corresponds to the no-template control. Lane M indicates the 100 bp DNA ladder.

A total of 47 blinded clinical specimens, comprising 31 samples from animals with pythiosis and 16 samples from animals without pythiosis, were evaluated using the LAMP-LFD and m-PCR assays (Figure 4). The disease status of all specimens had been previously confirmed by conventional culture and ITS sequence analysis. Using the LAMP-LFD assay, 28 of 31 pythiosis specimens were correctly identified as positive, whereas 14 of 16 non-pythiosis specimens were correctly identified as negative. This resulted in three false-negative and two false-positive results. In contrast, the m-PCR assay correctly identified 13 of 31 pythiosis specimens and all 16 non-pythiosis specimens, resulting in 18 false-negative results and no false-positive results. The LAMP-LFD assay achieved a sensitivity of 90.3% (95% CI: 74.3–98.0%), specificity of 87.5% (95% CI: 61.7–98.5%), PPV of 93.3% (95% CI: 79.2–98.1%), NPV of 82.4% (95% CI: 61.0–93.3%), and an overall accuracy of 89.4% (95% CI: 76.9–96.5%). In comparison, the m-PCR assay achieved a sensitivity of 41.9% (95% CI: 24.6–60.9%), specificity of 100% (95% CI: 79.4–100.0%), PPV of 100% (95% CI: 75.3–100.0%), NPV of 47.1% (95% CI: 39.7–54.5%), and an accuracy of 61.7% (95% CI: 46.4–75.5%). Agreement analysis showed that the LAMP-LFD assay had good diagnostic agreement with the reference standard (culture and ITS sequencing), with a Cohen’s kappa coefficient of 0.76 (95% CI: 0.57–0.95). In contrast, the m-PCR assay demonstrated only fair agreement, with a Cohen’s kappa coefficient of 0.32 (95% CI: 0.14–0.51).

## 4. Discussion

The continued emergence of pythiosis highlights the need for rapid, accurate, and user-friendly diagnostic methods to detect *P. insidiosum* [47]. In the present study, we developed a LAMP-LFD assay that combines the high analytical sensitivity of LAMP with the simplicity of an immunochromatographic lateral flow readout platform, which has been increasingly adopted for rapid molecular diagnostics [38,48]. Unlike our previously developed LAMP assay, which relied on agarose gel electrophoresis for amplicon detection and therefore required specialized equipment and trained personnel [21], the LFD format enables direct interpretation of amplification products within 15 min. Positive reactions are indicated by a clearly visible test line (Figure 1A), eliminating the need for post-amplification electrophoretic analysis. The LAMP-LFD assay can be completed in approximately 80 min, making it considerably faster than the m-PCR assay [42] and more practical for routine diagnostic use.

The LAMP-LFD assay demonstrated excellent analytical performance. All 51 *P. insidiosum* isolates representing clades I, II, and III were correctly detected, indicating complete inclusivity. Cross-reactivity was observed with three closely related *Pythium* species, namely *P. aphanidermatum*, *P. catenulatum*, and *P. rhizo-oryzae*, which have not generally been recognized as common causes of pythiosis in humans or animals [49]. This finding is consistent with previous analytical validation using the same ITS-targeted primer sequences and is likely attributable to the high sequence conservation of the ITS region among closely related *Pythium* species [22]. Therefore, the observed cross-reactivity most likely reflects limitations of the primer set rather than the LFD detection platform itself. The assay also demonstrated a detection limit of 10 fg of genomic DNA for all three *P. insidiosum* clades, representing a 1000- to 10,000-fold improvement in analytical sensitivity compared with m-PCR. Importantly, the incorporation of LFD did not compromise the analytical performance previously achieved using the same primer sequences [22], but instead provided a more convenient detection format [48,50].

The superior analytical sensitivity of the LAMP-LFD assay was reflected in its clinical performance. When evaluated using 47 blinded clinical specimens, the assay achieved a sensitivity of 90.3% and specificity of 87.5%, substantially outperforming m-PCR, which exhibited a sensitivity of only 41.9% despite maintaining perfect specificity. Notably, the LAMP-LFD assay detected more than twice as many pythiosis-positive specimens as the m-PCR assay and demonstrated markedly better agreement with the reference standard (culture and ITS sequencing) (κ = 0.76 versus 0.32). The high clinical sensitivity observed for LAMP-LFD is consistent with its substantially lower detection limit and likely reflects its ability to detect small quantities of pathogen DNA in clinical specimens, where low pathogen burden, uneven organism distribution, and DNA degradation may limit the performance of less sensitive amplification methods. In addition, unlike m-PCR, which requires post-amplification agarose gel electrophoresis for result visualization, the LAMP-LFD assay generates a direct visual readout, thereby simplifying workflow and reducing post-amplification processing requirements.

A key finding of the present study is that the LAMP-LFD assay addressed several limitations identified in our previously developed colorimetric LAMP (c-LAMP) assay [23]. Importantly, the same 47 blinded clinical specimens were evaluated in both studies [23], allowing direct comparison of the c-LAMP and LAMP-LFD platforms. Compared with c-LAMP, the LAMP-LFD assay demonstrated higher sensitivity (90.3% versus 83.9%), specificity (87.5% versus 68.8%), and overall accuracy (89.4% versus 78.7%), while reducing false-positive and false-negative results from five and five to two and three, respectively. The results suggest that direct amplicon detection by LFD provides a more reliable endpoint than indirect colorimetric detection. Similar observations have been reported in other LAMP-LFD systems, where direct dipstick detection improved result interpretation and reduced the subjective visual assessment associated with colorimetric assays [30,48]. Unlike c-LAMP, which infers amplification indirectly through HNB-mediated color changes resulting from Mg^2+^ depletion [23,51,52], the LAMP-LFD assay detects FITC-biotin double-labeled amplicons directly and generates a distinct test line upon successful amplification (Figure 1A). Consequently, weak amplification reactions that may yield subtle or ambiguous color shifts in c-LAMP can still be clearly identified by LFD. Furthermore, LFD results were consistently concordant with agarose gel electrophoresis, and no ambiguous or borderline reactions were observed.

Despite its promising performance, several discordant results were observed. Three pythiosis specimens produced false-negative results, possibly because of low pathogen burden, DNA degradation, or inhibitory substances in tissue extracts. Conversely, two non-pythiosis specimens yielded false-positive results. While the causes of these reactions could not be determined, possible explanations include contamination during specimen processing, nonspecific amplification, or the presence of low levels of target DNA not detected by the reference methods. Taken together, our previous studies and the present work demonstrate the progressive refinement of LAMP-based diagnostics for pythiosis, from gel-based detection [21] to colorimetric readout [22,23], and ultimately to LFD-based detection. Although the underlying amplification chemistry remained essentially unchanged [22,23], each successive platform addressed practical limitations of its predecessor, resulting in a highly sensitive assay with a rapid and easily interpretable visual endpoint.

Several limitations of this study should be acknowledged. First, the clinical evaluation was performed using a relatively small number of animal specimens, and performance in human pythiosis remains to be determined. Second, only three closely related *Pythium* species were included in the specificity assessment, and broader evaluation against additional oomycetes and other relevant microorganisms would provide a more comprehensive assessment of potential cross-reactivity. Third, the assay was evaluated using extracted genomic DNA, and future studies should investigate simplified sample-preparation approaches to further enhance field applicability. Finally, although quantitative PCR (qPCR) is recognized as a highly sensitive and specific molecular diagnostic method, it was not included in the present study because the primary objective was to evaluate the newly developed LAMP-LFD assay against m-PCR, which is routinely used in our laboratory. In addition, qPCR requires specialized instrumentation, fluorescent detection systems, and associated reagents that may not be readily available in resource-limited settings. Future studies directly comparing LAMP-LFD with qPCR platforms would provide an additional benchmark for assessing diagnostic performance and further strengthen confidence in the clinical utility of the assay.

In conclusion, a LAMP-LFD assay was successfully established for detecting *P. insidiosum*. The integration of the LFD platform enables rapid, direct, and easily interpretable visual detection while eliminating the need for time-consuming agarose gel electrophoresis. The assay demonstrated high analytical sensitivity, broad inclusivity across all tested *P. insidiosum* clades, and substantially improved clinical sensitivity compared with m-PCR. Furthermore, when evaluated using the same clinical specimens previously tested by c-LAMP, the LAMP-LFD format provided improved diagnostic performance and more reliable endpoint interpretation. Although additional validation using larger and more diverse specimen collections, particularly human specimens, is warranted, the findings demonstrate that direct LFD-based detection effectively overcomes several limitations of previous LAMP formats. Overall, the LAMP-LFD assay represents a practical and effective tool for rapid screening of pythiosis and may serve as an alternative molecular method in settings with limited access to qPCR or specialized molecular diagnostic infrastructure. However, given the observed false-positive results and cross-reactivity with several closely related *Pythium* species, LAMP-LFD should not be considered a standalone diagnostic test, and positive results should be interpreted in conjunction with clinical and laboratory findings and, when appropriate, confirmed using established reference methods.

## Figures and Tables

**Table 1:** Organisms used for genomic DNA preparation and analytical specificity testing of the loop-mediated isothermal amplification coupled with lateral flow dipstick (LAMP-LFD) and multiplex PCR (m-PCR) assays, including 51 *P. insidiosum* isolates, three closely related *Pythium* species, and 48 non-target fungal isolates.

Category	Organism (Genotype)	Number of Isolates	LAMP-LFD ^a^	m-PCR ^a^	Interpretation
Target organism	*Pythium insidiosum* (Clade I)	14	+	+	True detection
(*n* = 51)	*Pythium insidiosum* (Clade II)	23	+	+	True detection
	*Pythium insidiosum* (Clade III)	14	+	+	True detection
Related *Pythium* spp.	*Pythium aphanidermatum*	1	+	(**^_^**)	Cross-reactivity
(*n* = 3)	*Pythium catenulatum*	1	+	(^_^)	Cross-reactivity
	*Pythium rhizo-oryzae*	1	+	(^_^)	Cross-reactivity
Non-target fungi	*Acremonium* spp.	1	(^_^)	(^_^)	No cross-reactivity
(*n* = 48)	*Alternaria* spp.	5	(^_^)	(^_^)	No cross-reactivity
	*Aspergillus* spp.	3	(^_^)	(^_^)	No cross-reactivity
	*Aspergillus flavus*	3	(^_^)	(^_^)	No cross-reactivity
	*Aspergillus fumigatus*	1	(^_^)	(^_^)	No cross-reactivity
	*Aspergillus glaucus*	1	(^_^)	(^_^)	No cross-reactivity
	*Aspergillus niger*	1	(^_^)	(^_^)	No cross-reactivity
	*Aspergillus terreus*	1	(^_^)	(^_^)	No cross-reactivity
	*Chaetomium* spp.	2	(^_^)	(^_^)	No cross-reactivity
	*Chrysosporium* sp.	1	(^_^)	(^_^)	No cross-reactivity
	*Cladophialophora bantiana*	1	(^_^)	(^_^)	No cross-reactivity
	*Cladosporium* sp.	1	(^_^)	(^_^)	No cross-reactivity
	*Curvularia* spp.	6	(^_^)	(^_^)	No cross-reactivity
	*Daldinia* sp.	1	(^_^)	(^_^)	No cross-reactivity
	*Fusarium* spp.	3	(^_^)	(^_^)	No cross-reactivity
	*Histoplasma capsulatum*	1	(^_^)	(^_^)	No cross-reactivity
	*Microsporum gypseum*	1	(^_^)	(^_^)	No cross-reactivity
	*Neoscytalidium* sp.	1	(^_^)	(^_^)	No cross-reactivity
	Non-sporulating fungus	1	(^_^)	(^_^)	No cross-reactivity
	*Rhizopus* sp.	1	(^_^)	(^_^)	No cross-reactivity
	*Scedosporium apiospermum*	1	(^_^)	(^_^)	No cross-reactivity
	*Syncephalastrum* sp.	1	(^_^)	(^_^)	No cross-reactivity
	*Talaromyces marneffei*	5	(^_^)	(^_^)	No cross-reactivity
	*Trichoderma* sp.	1	(^_^)	(^_^)	No cross-reactivity
	*Trichophyton mentagrophytes*	2	(^_^)	(^_^)	No cross-reactivity
	*Trichophyton rubrum*	2	(^_^)	(^_^)	No cross-reactivity

Footnotes: ^a^ The symbol ‘+’ indicates a positive amplification reaction, whereas ‘(^_^)’ indicates a negative amplification reaction.

## Data Availability

The original contributions presented in this study are included in the article. Further inquiries can be directed to the corresponding author.

## Supplementary Materials

The following supporting information can be downloaded at: https://www.mdpi.com/article/10.3390/jof12080589/s1, Figure S1. Analytical specificity of the m-PCR assay for the detection of *P. insidiosum*. Lanes 1–51 correspond to *P. insidiosum* isolates representing clades I–III. Lanes 52–54 correspond to the closely related species *P. aphanidermatum*, *P. catenulatum*, *and P. rhizo-oryzae*, respectively. Lanes 55–102 correspond to non-target fungal isolates listed in Table 1. Lane 103 corresponds to the no-template control. Lane M indicates the 100 bp DNA ladder.
