# Supplementary material for: Rapid Visual Detection of Pythium insidiosum by LAMP-LFD
Source: J Fungi (Basel). 2026 Aug 8;12(8):589. doi: 10.3390/jof12080589 (PMC13514608; doi:10.3390/jof12080589)
Supplement: Supplementary file 1 [file jof-12-00589-s001.zip › jof-4459789-supplementary.pdf]

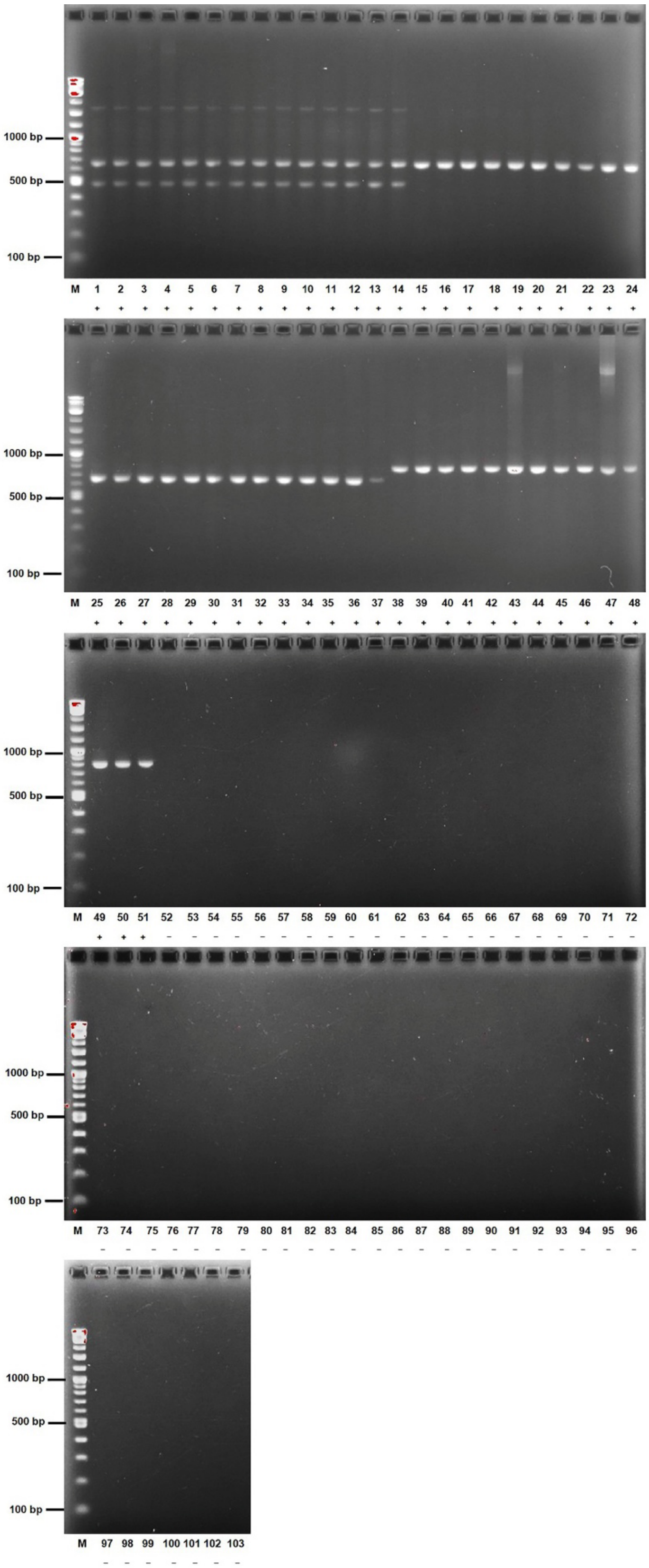

Supplementary Figure S1. Analytical specificity of the m-PCR assay for the detection of *P. insidiosum*. Lanes 1–51 correspond to *P. insidiosum* isolates representing clades I–III. Lanes 52–54 correspond to the closely related species *P. aphanidermatum*, *P. catenulatum*, *and P. rhizo-oryzae*, respectively. Lanes 55–102 correspond to non-target fungal isolates listed in Table 1. Lane 103 corresponds to the no-template control. Lane M indicates the 100-bp DNA ladder.
